# Terrestrial dimethyl sulfide: Template for astrobiology

**DOI:** 10.6026/973206300211668

**Published:** 2025-06-30

**Authors:** Paul Shapshak, Jean Schneider, Beata Casanas, Charurut Somboonwit, John T. Sinnott

**Affiliations:** 1 Department of Internal Medicine, Division of Infectious Diseases and International Health, Morsani College of Medicine, University of South Florida, Tampa, Florida - 33606 USA; 2 Observatoire de Paris, University of Paris, Paris, France

**Keywords:** Dimethyl sulfide (DMS), dimethyl-sulfonio-propionate (DMSP), terrestrial marine microbial metabolism, viruses, sulfur cycling pathways, climate feedback, atmospheric chemistry, biosignatures, vegetation reflectance, astrobiology, asteroseismology, Hycean sub-Neptune exoplanets, mid-infrared transmission spectra, James Webb Space Telescope MIRI instrument

## Abstract

Dimethyl sulfide (DMS) is a volatile breakdown product of dimethyl-sulfonio-propionate (DMSP). DMS is synthesized by marine phytoplankton, plays a central role in Earth's sulfur cycle and has growing relevance in astrobiology. DMS formation is mediated by complex microbial, viral, enzymatic and photochemical processes that reflect dynamic interactions within marine ecosystems. Bacterial pathways responsible for DMSP degradation are genetically diverse and ecologically widespread, while viral lysis of phytoplankton contributes to release and recycling of sulfur compounds. Once released into the atmosphere, DMS contributes to sulfate aerosol formation, influencing cloud condensation and planetary albedo - an example of biogeochemical feedback between ocean life and climate. This report reviews terrestrial DMS production, including microbial, viral and abiotic contributions. Given its volatility, detectability and potential biological origin, DMS is considered a promising biosignature candidate in the search for life on exoplanets.

## Background:

Dimethyl sulfide (DMS) is a sulfur-containing compound that terrestrially is primarily produced through biological processes. It originates from the enzymatic cleavage of dimethyl-sulfonio-propionate (DMSP), a sulfur-based osmoprotectant synthesized by marine phytoplankton and algae. The recent claim of DMS detection in the atmosphere of the exoplanet K2-18b K2-18 b [1] has raised the possibility of astrobiological activity. However, any assertion of extraterrestrial life requires careful consideration of alternative synthetic pathways, which must be thoroughly evaluated before exobiological inferences can be made [1, 2, 3-4]. Madhusudhan *et al.* reported the detection of DMS and dimethyl disulfide (DMDS) in the atmosphere of the exoplanet K2-18b, using mid-infrared transmission spectra from the James Webb Space Telescope's MIRI instrument [1]. On Earth, DMS is produced by marine phytoplankton [5]. Its detection in a distant exoplanetary atmosphere may be a potential biosignature. K2-18b is a sub-Neptune planet approximately 8.6 times Earth's mass and 2.6 times its radius, orbits within the habitable zone of the M-dwarf star K2-18, located about 124 light-years away, in the constellation, Leo. It is considered a "Hycean" world - possessing a hydrogen-rich atmosphere and possibly global oceans [3, 6 and 7]. The DMS signal was detected at a minimum abundance of ~10 parts per million, with ~3σ significance, suggesting a possible - but not definitive - biosignature [1]. Further spectroscopic observations are required to confirm the signal and further studies are necessary to rule out non-exobiological sources. Therefore, it is of interest to report that DMS and the sulfur cycle on planet Earth are models or templates for possible biosignatures on some exoplanets.

## Terrestrial biological production of dimethyl sulfide (DMS):

On Earth, DMS is closely tied to the marine sulfur cycle. DMSP, the primary precursor to DMS, is produced by phytoplankton species such as Emiliania huxleyi and Phaeocystis spp [8, 9]. DMSP is released into seawater following cell death, viral lysis, or zooplankton grazing, making it accessible to marine bacteria [10]. These bacteria-including Roseobacter, Pelagibacter ubique (SAR11) and Marinobacter spp.-catabolize DMSP via two main routes [11, 12]. Cleavage Pathway: DMSP lyase enzymes convert DMSP into volatile DMS and acrylate. This pathway is ecologically important, as DMS escapes into the atmosphere and contributes to cloud formation and climate regulation [5, 13]. Demethylation Pathway: DMSP is broken down into methanethiol, incorporating sulfur into microbial biomass without releasing DMS [11]. Additionally, DMS can be oxidized to form dimethyl sulfoxide (DMSO) via photochemical reactions or bacterial processes [14, 15]. DMSO represents a secondary product in the marine sulfur cycle. Notably, abiogenic DMS has been detected in comets, indicating that exoplanetary DMS could potentially arise from cometary impacts rather than biological activity [16].

## DMSP and DMS cycling in marine environments:

The biogeochemical cycling of DMSP and DMS is a major process in marine ecosystems, with implications for climate regulation. DMSP, produced by marine phytoplankton, is a precursor to DMS-a compound that enhances cloud condensation and planetary albedo [5]. Production and turnover of DMSP are influenced by light availability, nutrient concentrations and microbial interactions [9]. Phytoplankton contributes to DMS formation not only through DMSP cleavage but also via the enzymatic reduction of DMSO [15]. Viral lysis of phytoplankton further enhances the sulfur cycle by increasing DMSP release into microbial communities [17]. The inter-conversion of DMS and DMSO, as details by Hatton *et al.* [14] underscores the dynamic nature of the marine sulfur cycle.

## Microbial catabolism and genetic regulation of DMSP:

Microbial metabolism is central to DMSP transformation. Curson *et al.* [1] reviewed the key bacterial enzymes and genes responsible for DMS and methanethiol production. Later, Curson *et al.* [12] identified genes involved in bacterial DMSP biosynthesis, revealing evolutionary connections with phytoplankton. Studies by Howard *et al.* [19] and Moran *et al.* [10] demonstrated the wide distribution and genetic specialization of DMSP-catabolizing bacteria in marine surface waters. Reisch *et al.* [11] described a novel assimilation pathway for DMSP in diverse marine bacteria. Sun *et al.* [20] demonstrated that Pelagibacter species can metabolize DMSP into both DMS and methanethiol, reflecting their dual roles in sulfur cycling.

## Bacterial metabolic pathways of DMS production:

Marine bacteria, especially within the Alpha and Gammaproteobacteria, transform DMSP into DMS under both aerobic and anaerobic conditions via DMSP lyase-mediated cleavage [18]. These include Roseobacter, Pelagibacter ubique and Marinobacter spp. [19, 20]. While DMS is not a major metabolic substrate for these microbes, the acrylate produced in this pathway may be further metabolized. Ecologically, bacterial DMS production has global significance. Once released into the atmosphere, DMS participates in climate-modulating processes. Its potential detection in distant planetary atmospheres strengthens its potential as a biosignature gas [21]. Table 1 (see PDF) shows a summary of key sulfur microbial pathways.

## Marine viruses and ecosystem dynamics:

Marine viruses regulate microbial communities and influence biogeochemical cycles, particularly within the phycosphere-the microscale zone surrounding phytoplankton cells. Seymour *et al.* [22] described this niche as a hotspot for viral-microbial interactions that influence nutrient dynamics and signal exchange. Viral lysis of phytoplankton, particularly by members of the Phycodnaviridae family, results in the release of DMSP [17, 22]. Bacteria then metabolize this DMSP via both cleavage (producing DMS and acrylate) and demethylation pathways [23]. As a result, viral lysis events can significantly amplify DMS emissions, influencing cloud condensation and contributing to climate feedback via sulfur oxidation products like sulfuric and methanesulfonic acids [5].

## Atmospheric chemistry and climate feedback:

DMS released from the ocean is a key precursor to sulfate aerosols, which promotes cloud condensation nuclei and influence Earth's albedo. Andreae and Crutzen [13] examined this relationship, linking atmospheric aerosols with their marine biogenic sources. The "CLAW hypothesis" proposed by Charlson *et al.* [5] illustrates how phytoplankton-derived DMS could affect global climate by modulating cloud properties and Earth's radiation balance.

## Biosignature gases and astrobiological relevance:

DMS is among the most promising biosignature gases for detecting life beyond Earth. Seager *et al.* [21, 24] proposed that volatile organosulfur compounds, including DM, can persist in hydrogen-rich atmospheres for long durations. Schwieterman *et al.* [25] expanded this framework, analyzing a wider array of biosignature gases and emphasizing the necessity of contextual planetary data for accurate interpretation.

## Conclusions and implications for astrobiology:

This review underscores the intricate connections between DMSP/DMS cycling, microbial ecology, atmospheric chemistry and climate regulation. The pathways of DMSP metabolism-mediated by bacteria and influenced by viral dynamics-illustrates the complex interplay driving sulfur fluxes in marine systems. The detection of DMS in exoplanetary atmospheres may point to sulfur-based biochemistry akin to Earth's oceans. This includes the potential existence of DMSP-like precursors and microbial redox processes operating under variable oxygen conditions [24, 25-26]. DMS and related sulfur compounds thus hold promise as candidate biosignatures in the search for life, particularly on ocean-bearing exoplanets. Nevertheless, abiotic production mechanisms, such as cometary delivery, must be carefully considered alongside exobiological interpretations. A way to reinforce the interpretation of DMS detection as a biosignature is to combine it with two other potential biosignatures. The first is the detection of the O2 absorption band at 760 nm in the atmosphere of the planet. That is feasible with the Hubble telescope STIS spectrograph and the JWST NearSpec spectrograph. The second potential biosignature is an analog of the "vegetation red edge" at the planet surface. When the planet atmosphere is transparent, one can detect spectral features of the planet surface in direct imaging of the planet that cannot be due to mineral species. On Earth it is the case for the 725-1200 nm band in the reflectance spectrum common to all vegetation. An example of a terrestrial vegetation reflectance spectrum is shown in Figure 1 (see PDF). On another planet, the products of a photosynthetic activity ("generalized vegetation") are likely to have other reflectance spectral features than terrestrial vegetation [27, 28, 29-30]. To avoid confusion with mineral spectra one then has to ensure that they are not presenting in any reflectance spectrum [31] available for example at the USGS database. The latter approach will required high sensitivity and high angular resolution telescopes, like the Planetary Camera Systems at the coming E-ELT and the Habitable Worlds Observatory [32- 33].
